# Characteristics and biomarkers of patients with central nervous system infection admitted to a referral hospital in Northern Vietnam

**DOI:** 10.1186/s41182-021-00322-2

**Published:** 2021-05-21

**Authors:** Cuong Chi Ngo, Shungo Katoh, Futoshi Hasebe, Bhim Gopal Dhoubhadel, Tomoko Hiraoka, Sugihiro Hamaguchi, Anh Thi Kim Le, Anh Thi Hien Nguyen, Anh Duc Dang, Chris Smith, Lay-Myint Yoshida, Cuong Duy Do, Thuy Thi Thanh Pham, Koya Ariyoshi

**Affiliations:** 1 Department of Clinical Medicine, Institute of Tropical Medicine, Nagasaki University, Nagasaki, Japan; 2 Nagasaki University Graduate School of Biomedical Sciences, Nagasaki, Japan; 3 Center for Tropical Diseases, Bach Mai Hospital, Hanoi, Vietnam; 4 Department of General Internal Medicine and Clinical Infectious Diseases, Fukushima Medical University, Fukushima, Japan; 5 Department of General Internal Medicine and Infectious Diseases, Kita-Fukushima Medical Center, Fukushima, Japan; 6 Vietnam Research Station, Institute of Tropical Medicine (NEKKEN), Nagasaki University, Nagasaki, Japan; 7 School of Tropical Medicine and Global Health, Nagasaki University, Nagasaki, Japan; 8 Department of Respiratory Infections, Institute of Tropical Medicine (NEKKEN), Nagasaki University, Nagasaki, Japan; 9 Department of General Internal Medicine, Nagasaki Rosai Hospital, Nagasaki, Japan; 10 Department of General Internal Medicine, Fukushima Medical University, Fukushima, Japan; 11 National Institute of Hygiene and Epidemiology, Hanoi, Vietnam; 12 Department of Clinical Research, London School of Hygiene and Tropical Medicine (LSHTM), London, England; 13 Department of Pediatric Infectious Diseases, Institute of Tropical Medicine (NEKKEN), Nagasaki University, Nagasaki, Japan; 14 The Partnership for Health Advancement in Vietnam (HAIVN), Hanoi, Vietnam

**Keywords:** CNS infection, Meningitis, *S. suis*, HSV, Tubercular meningitis, Biomarker, ADA, Vietnam

## Abstract

**Background:**

Laboratory facilities for etiological diagnosis of central nervous system (CNS) infection are limited in developing countries; therefore, patients are treated empirically, and the epidemiology of the pathogens is not well-known. Tubercular meningitis is one of the common causes of meningitis, which has high morbidity and mortality, but lacks sensitive diagnostic assays. The objectives of this study were to determine the causes of meningitis in adult patients by using molecular assays, to assess the risk factors associated with them, and to explore whether biomarkers can differentiate tubercular meningitis from bacterial meningitis.

**Methods:**

We conducted a cross-sectional study in the Department of Infectious Diseases, Bach Mai Hospital, Hanoi, Vietnam, from June 2012 to May 2014. All patients who were 16 years old and who had meningoencephalitis suggested by abnormal cerebrospinal fluid (CSF) findings (CSF total cell >5/mm3 or CSF protein 40 mg/dL) were included in the study. In addition to culture, CSF samples were tested for common bacterial and viral pathogens by polymerase chain reaction (PCR) and for biomarkers: C-reactive protein and adenosine deaminase (ADA).

**Results:**

Total number of patients admitted to the department was 7506; among them, 679 were suspected to have CNS infection, and they underwent lumbar puncture. Five hundred eighty-three patients had abnormal CSF findings (meningoencephalitis); median age was 45 (IQR 3158), 62.6% were male, and 60.9% were tested for HIV infection. Among 408 CSF samples tested by PCR, out of them, 358 were also tested by culture; an etiology was identified in 27.5% (*n*=112). *S. suis* (8.8%), *N. meningitis* (3.2%), and *S. pneumoniae* (2.7%) were common bacterial and HSV (2.2%), Echovirus 6 (0.7%), and Echovirus 30 (0.7%) were common viral pathogens detected. *M. tuberculosis* was found in 3.2%. Mixed pathogens were detected in 1.8% of the CSF samples. Rural residence (aOR 4.1, 95% CI 1.214.4) and raised CSF ADA (10 IU/L) (aOR 25.5, 95% CI 3.1212) were associated with bacterial meningitis when compared with viral meningitis; similarly, raised CSF ADA (10 IU/L) (aOR 42.2, 95% CI 2.0882) was associated with tubercular meningitis.

**Conclusions:**

Addition of molecular method to the conventional culture had enhanced the identification of etiologies of CNS infection. Raised CSF ADA (10 IU/L) was strongly associated with bacterial and tubercular meningitis. This biomarker might be helpful to diagnose tubercular meningitis once bacterial meningitis is ruled out by other methods.

**Supplementary Information:**

The online version contains supplementary material available at 10.1186/s41182-021-00322-2.

## Background

Central nervous system (CNS) infection is ten times more common in developing countries than in developed countries, where clinical diagnosis and empiric treatment without etiological confirmation is the main way of management [1, 2]. The CNS infection in the tropical climates is complex because a wide range of pathogens can cause it, including arboviruses, mycobacterium, fungi, protozoa, and helminths, besides pathogens commonly seen in developed countries [3]. The incidence and etiology of CNS infection vary with time, geographical area, age of patients, underlying diseases, and vaccination status of the population [4].

In Vietnam, mortality and morbidity due to CNS infections are high; case fatality rate was 812% (21 deaths out of 262 in a study and 73 deaths out of 617 in another study), and residual disability was reported in 1030% of survived patients [5, 6]. Few studies have investigated the etiologies of CNS infection and showed that *Streptococcus suis* and *Herpes simplex* virus are the main bacterial (2450%) and viral (3.54%) causes of meningoencephalitis syndrome [5, 6]. However, 5075% of the cases did not have a confirmed etiological diagnosis. The low proportion of confirmed diagnoses can be because of previous antibiotic use, natural clearance of virus, limited number of pathogens targeted in molecular assays, late lumbar puncture, and low sensitivity of diagnostic assays [5, 6]. Moreover, a relatively high frequency of tubercular meningitis (TBM) is attributed to the complexity of CNS infection in developing countries, including Vietnam. In Vietnam, TBM is confirmed in 2.66.0% (9/352-34/617) of CNS infections [5, 6]. TBM requires specific anti-TB drugs for a longer period than conventional bacterial meningitis, and discrimination of TBM from bacterial meningitis by clinical features alone is difficult and often impossible [7]. Many studies have shown that delays in diagnosis, which leads to delayed treatment, are a major contributing factor of high mortality of TBM [8,10]. TBM is diagnosed by culture and isolation of *M. tuberculosis* from CSF; this conventional method is slow and insensitive to aid clinician to decide on time [11]. Novel highly sensitive diagnostic assays are in need for TBM. CSF ADA is a biomarker, which has been investigated for the potential use for the diagnosis of TBM; however, more clinical studies for its validation are needed [12,14].

As the epidemiological and etiological data of CNS infections in Northern Vietnam are limited, we conducted this study to identify the common etiologies of CNS infections, including *M. tuberculosis* by using molecular methods in CSF samples of adult meningoencephalitis patients. We aimed to analyze the risk factors associated with common etiologies of CNS infections and their association with biomarkers.

## Methods

### Study design, enrollment criteria, and sample collection

This is a hospital-based cross-sectional study, which was carried out in the department of Infectious Diseases of Bach Mai Hospital, Hanoi, Vietnam. This hospital is the largest tertiary referral medical center with the patient coverage of all the areas of northern Vietnam. The infectious disease ward had 120 beds and had approximately 4000 patient admissions per year when this study was conducted. The study was conducted between June 2012 and May 2014. All patients, who were admitted to the infectious disease ward, were eligible to the study if they met the following inclusion criteria: (1) age of 16 year; (2) meningoencephalitis defined by abnormal cerebrospinal fluid (CSF) findings: CSF total cell >5/mm3 or CSF protein 40 mg/dL. All patients were managed by physicians in the department with routine clinical care.

### Microbiological investigations

#### Sample collection and storage

About 2 to 5 mL CSF and 10 mL blood were collected. One milliliter CSF and 5 mL blood were separated and immediately stored at 20 C for PCR. Remaining samples were used for routine tests; microscopy, including Gram staining and ZN staining, and culture. Later, the samples for PCR were transferred to NIHE and stored at 80 C till the DNA was extracted.

#### Blood and CSF culture

Blood and CSF cultures were performed in the Microbiology Laboratory Department, Bach Mai Hospital, using standard culture methods. Blood culture was performed using BD BACTEC 9240 blood culture system (Becton Dickinson, USA). CSF samples were cultured on blood and chocolate agar plates and inoculated at 37C in 5% CO_2_ for 96 h before declaring negative.

#### Molecular methods

DNA was extracted from CSF samples by using Qiagen kit following the manufacturers protocol within 1 year of the end of study period, and polymerase chain reaction (PCR) was performed. Real-time PCR from PCR ROCHE Diagnostics was used to detect *Mycobacterium tuberculosis* and *Herpes simplex virus*. These techniques were done in the Microbiology Laboratory Department of Bach Mai Hospital.

We applied multiplex PCR to identify *Streptococcus pneumoniae*, *Neisseria meningitidis*, and *Haemophilus influenza* and a single PCR for *Streptococcus suis*, following previously published methods [15, 16]. For detection of viral pathogens, real-time PCR and virus isolation methods were used [17,20].

#### Serological methods

*Cryptococcus neoformans* capsular polysaccharide antigen was tested in all CSF samples by using the Eiken test (Eiken, Tokyo, Japan).

#### Tests for biomarkers

In addition to the routine investigations of C-reactive protein (CRP) and procalcitonin (PCT) in blood, we measured the level of adenosine deaminase (ADA) in CSF. ADA was measured by the SEIKEN ADA II test kit (Denka, Tokyo, Japan).

#### Data collection

Epidemiological and clinical, including hospital and laboratory, data were collected from patients medical charts by investigators and trained research assistants in a standard questionnaire in the paper format. The data later were transferred to Microsoft Access.

### Statistical analysis

Demographic and clinical characteristics and laboratory findings of bacterial meningitis, TB meningitis, and viral meningoencephalitis were compared. The categorical variables were presented as proportions and compared by the Chi-squared test or Fisher exact test, and the continuous variables were presented as the medians (IQR) and compared by using Kruskal-Wallis test. In multivariate analysis, variables with *P* value less than 0.2 in the univariate analysis, and age and sex were included. Using the backward stepwise method, the results of the final model were presented. *P* value <0.05 was regarded as the statistical significance. STATA version 14 (StataCorp LP, College Station, TX) was used for statistical analysis.

### Ethical approval

This study was approved by the Institutional Review Boards of Bach Mai Hospital, Hanoi, Vietnam, and Institute of Tropical Medicine, Nagasaki University, Nagasaki, Japan. An informed written consent was obtained from all the patients prior to the enrollment. A parent or guardian gave the consent in cases of unconscious patients.

## Results

### Characteristics of the study population

**Fig. 1:**
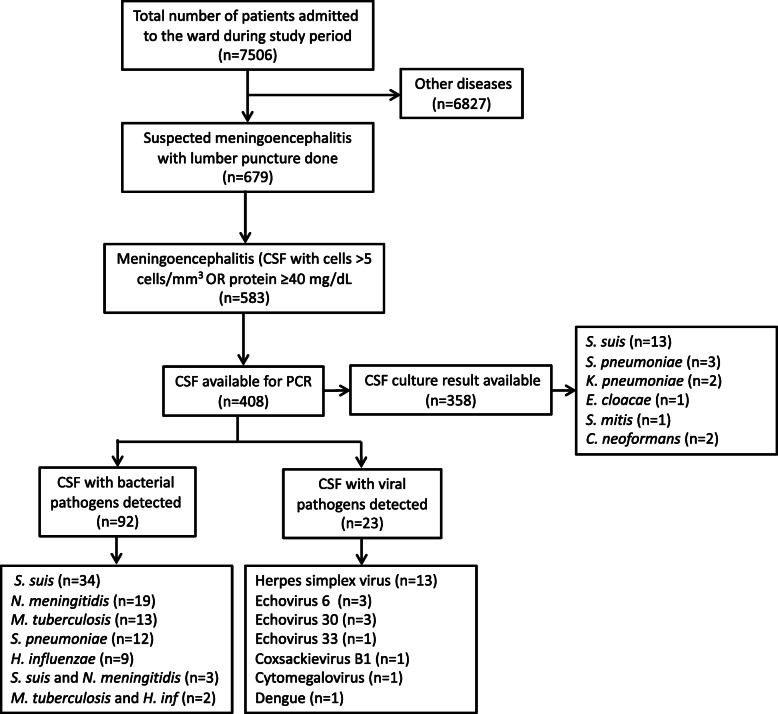
Flowchart of the study showing the number of patients admitted to the ward during the study period to the detection of various pathogens in CSF

**Fig. 2:**
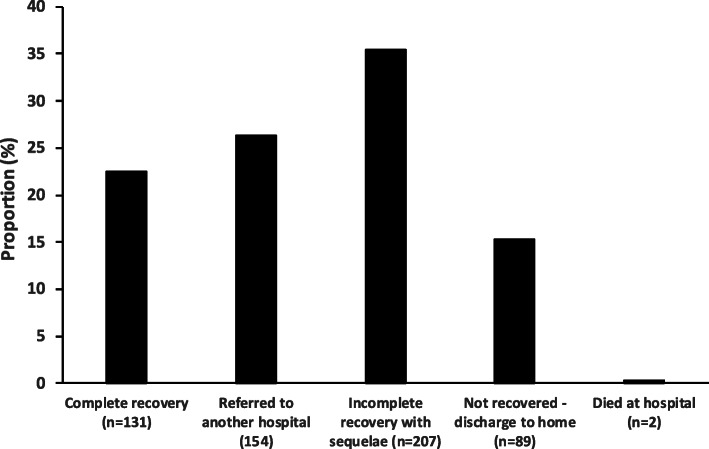
Bar graph showing the outcomes of the patients with CNS infections (*n*=583)

The total number of patients admitted to the ward during the study period was 7506. Among these patients, 679 had symptoms and signs of CNS infection. Lumbar punctures were done in these suspected CNS infection patients, and 583 patients had raised cells (>5 cells/mm3) or increased protein (40 mg/dL) (Fig.1). Epidemiological and clinical characteristics of these patients are shown in Table1. Briefly, 62.6% were male; median age was 45 years; 69.1% were from rural areas. There was a strong association between living in rural areas and exposure to pig (*p*<0.001). In terms of clinical characteristics, 6.9% had diabetes, 6.4% were HIV positive, and 26.6% had antibiotics before admission to the hospital. Common presenting symptoms were headache (84.7%), fever (>38C) (38.8%), and loss of consciousness (37.9%); similarly, common physical findings were neck stiffness (38.4%), low GCS score (8 to 14 in 29.6% and <8 in 2.4%), rash (12.0%), and hepatomegaly (10.8%). Among the patients with CNS infection, 2 (0.3%) died at the hospital, and 89 (15.3%) were discharged to home in a comatose state (not recovered). The details of the outcomes are shown in Fig.2. CSF samples were available from 408 patients for PCR, and culture results were available from 358 patients (Fig.1).

**Table 1:** Epidemiological and clinical characteristics of study population

Characteristics	*N*=583 (%)
Age, median (p25-p75)	45 (3158)
Male sex	365 (62.6)
Geography of residence	
Flatland	444 (76.2)
Mountain	69 (11.8)
Coastal area	70 (12.0)
Place of residence	
Urban	180 (30.9)
Rural	403 (69.1)
Exposure to animals, pig	15 (2.6)
Immunocompromised state	
Diabetes	40 (6.9)
Cirrhosis	13 (2.2)
Cancer	6 (1.0)
HIV	37 (6.4)
Antibiotic use before hospitalization	155 (26.6)
Referred from other hospitals	386 (66.2)
Fever (>38C)	224 (38.8)
Duration of fever at admission, <7 days	412 (70.7)
Headache	494 (84.7)
History of convulsion	38 (6.5)
History of loss of consciousness	221 (37.9)
Neck stiffness	224 (38.4)
Rash	70 (12.0)
Hypoxemia	34 (5.8)
Hypotension	4 (0.7)
Hepatomegaly	63 (10.8)
Splenomegaly	10 (1.7)
Glasgow Coma Scale	
8 to 14	171 (29.6)
<8	14 (2.4)
Outcomes	
Not recovered-discharged to home	89 (15.3)
Death at hospital	2 (0.3)

We found that 355 patients (60.9%) of 583 CNS-infected patients were tested for HIV status. We compared the characteristics of HIV tested and not tested patients (Table S1). We found that age less than 45 years, male sex, hepatomegaly, referral to another hospital, and hospital admission duration over 7 days were independently associated with HIV-tested patients.

### Distribution of etiologic pathogens

By PCR, we detected bacterial pathogens in 92 samples and viral pathogens in 23 samples among 408 CSF samples (Fig.1). Among those whose culture results were available, we found that 22 samples yielded a bacterial or a fungal pathogen. Combining both PCR and culture results, we found an etiological cause of meningitis in 112 patients out of 408 who had abnormal CSF findings. Table2 shows the distribution of these pathogens. *S. suis* (8.8%) was the most common bacterial isolate, which was followed by *N. meningitidis* (3.2%) and *S. pneumoniae* (2.7%). Among viral pathogens, *HSV* (2.2%) was found to be most common, and it was followed by *Echovirus 6* (0.7%) and *Echovirus 30* (0.7%). *M. tuberculosis* was detected in 13 cases (3.2%). In CSF of seven patients, two or more pathogens were detected*. C. neoformans* was detected in a total of three cases (2 from culture and 1 from cryptococcal antigen test of CSF). Mixed pathogens were detected in seven CSF samples.

**Table 2:** Distribution of pathogens detected in suspected cases of meningoencephalitis

Pathogens detected	*N*=408 (%)
Bacteria	
*S. suis*	36 (8.8)
*N. meningitidis*	13 (3.2)
*S. pneumoniae*	11 (2.7)
*H. influenzae*	7 (1.7)
*K. pneumoniae*	2 (0.5)
*E. cloacae*	1 (0.3)
*S. mitis*	1 (0.3)
Virus	
HSV	9 (2.2)
Echovirus 6	3 (0.7)
Echovirus 30	3 (0.7)
CMV	1 (0.3)
Coxsackievirus B1	1 (0.3)
Echovirus 33	1 (0.3)
Dengue	1 (0.3)
*M. tuberculosis*	13 (3.2)
Fungus	
*C. neoformans*	2 (0.5) ^a^
Mixed	
*M. tuberculosis* and *H. influenzae*	2 (0.5)
*S. suis* and *N. meningitidis*	2 (0.5)
*S. suis* and *N. meningitidis* and HSV	1 (0.3)
*N. meningitidis* and HSV	2 (0.5)
Negative in PCR or culture	296 (72.5)

^a^One more case of *C. neoformans* was detected by antigen test

Based on a single identified etiological pathogen, we classified common CNS infection into bacterial meningitis (*N*=73), tuberculous meningitis (*N*=13), viral meningitis (*N*=19), and fungal meningitis (*N*=3) (Table3).

**Table 3:** Demographic and clinical characteristics of viral, bacterial, and tubercular meningitis

Characteristics	Viral meningitis *N*=19 (%)	Bacterial meningitis *N*=73 (%)	Tubercular meningitis *N*=13 (%)	*P* value
Age, median (p25-p75)	31 (2251)	50 (3260)	50 (3069)	0.048
Male sex	12 (63.2)	55 (75.3)	7 (53.9)	0.218
Place of residence, rural	7 (36.9)	50 (68.5)	10 (76.9)	0.022
Exposure to animal, pig	0 (0.0)	7 (9.6)	0 (0.0)	0.379
Immunocompromised state^a^	1 (5.3)	10 (13.7)	4 (30.8)	0.146
Duration of fever at admission, 7 days	6 (31.6)	26 (35.6)	7 (53.9)	0.391
Headache	16 (84.2)	64 (87.7)	11 (84.6)	0.738
History of loss of consciousness	11 (57.9)	39 (53.4)	9 (69.2)	0.561
Neck stiffness	6 (31.6)	41(56.2)	6 (46.2)	0.153
Glasgow Coma Scale (*n*=102)				
8 to 14	10 (55.6)	28 (39.4)	8 (61.5)	0.405
<8	0 (0.0)	6 (8.5)	0 (0.0)	
Abnormal CT/MRI brain (*n*=62)	9 (75.0)	22 (56.4)	4 (36.4)	0.201
HIV positive	1 (5.3)	1 (1.4)	2 (15.4)	0.049
Lab findings				
White blood cells (10^9^/L), median	13.0	13.8	7.8	0.038
Neutrophil count (10^9^/L), median	8.8	11.6	6.8	0.035
Raised liver enzymes (AST>40 or ALT>35 U/L) (*n*=101)	9 (47.4)	43 (62.3)	5 (38.5)	0.191
Raised blood urea (>7.1 mmol/L) (*n*=103)	2 (10.5)	16 (22.5)	5 (38.5)	0.181
Raised CRP (1 mg/dL) (*n*=95)	7 (43.8)	50 (74.6)	7 (58.3)	0.047
Raised procalcitonin (0.5 ng/mL) (*n*=38)	3 (33.3)	16 (57.1)	0 (0.0)	0.269
CSF analysis				
White blood cells (/L), median	70	525	250	0.002
Neutrophil count (/L), median	33	297	175	0.003
Protein (g/L), median	0.8	2.1	1.7	0.001
CSF/blood glucose ratio, median	60.4	26.1	36.1	0.001
ADA (IU/L), median (*n*=103)	3.2	14.3	13.0	0.001
Outcome				
Complete recovery	6 (31.6)	16 (21.9)	0 (0.0)	0.001
Referred to another hospital	4 (21.0)	16 (21.9)	11 (84.6)	
Incomplete recovery	8 (42.1)	30 (41.1)	1 (7.7)	
Not recovered-discharged to home	1 (5.3)	11 (15.0)	1 (7.7)	
Death at hospital	0 (0.0)	0 (0.0)	0 (0.0)	

Data are presented as No. (%) for categorical variables and median for continuous variables. *P* values are from Chi-squared tests for categorical variables and Kruskal-Wallis test for continuous variables

^a^This includes diabetes mellitus, liver cirrhosis, HIV positive, renal diseases, cancer, liver diseases, and chronic alcoholic diseases

### Comparison of viral, bacterial, and tubercular meningitis

Demographic and clinical characteristics of viral, bacterial, and tubercular meningitis are compared in Table3. Patients with viral meningitis tended to be younger than bacterial or tubercular meningitis; the median age was 31 years in viral meningitis versus 50 years in other types. The proportions of patients from the rural areas were significantly higher in bacterial and tubercular meningitis than in viral meningitis. Although there was no significant association between bacterial meningitis and exposure to pig, out of 39 patients infected with the most common bacterial pathogen *S. suis*, 7 (17.9%) patients were exposed to pig, and this association was highly significant (*P*<0.001). In laboratory findings, WBC count was higher in viral and bacterial meningitis than tubercular meningitis, and the proportion of raised CRP levels was found higher in bacterial and tubercular meningitis than viral meningitis. Similarly, the CSF analysis showed higher WBC count, protein level, and ADA levels in bacterial and tubercular meningitis than viral meningitis. Regarding the outcomes, most tubercular meningitis patients were referred to TB hospital, so we could not follow their treatment outcome from there. When compared between viral and bacterial meningitis patients, the proportion of complete recovery was high in viral meningitis (31.6% vs 21.9%), and the proportion of not recovered-discharged to home patients was high in bacterial meningitis (5.3% vs 15.0%). Both these findings suggested the severe outcomes were more common in bacterial meningitis than that of viral meningitis.

Table4 shows the univariate and multivariate analyses of bacterial meningitis versus viral meningitis and tubercular meningitis versus viral meningitis. Age 40 years, rural residence, raised CRP, and all parameters in CSF analysis (raised WBC count, neutrophil count, protein, and ADA; and low CSF/blood glucose ratio) were associated with bacterial meningitis in the univariate analysis. In multivariate analysis, we found that rural residence and raised ADA (10 IU/L) were independently associated with bacterial meningitis as compared with viral meningitis. Similarly, rural residence, raised CSF protein, lower CSF/blood glucose ratio, and raised ADA level were associated with tubercular meningitis in the univariate analysis; however, only raised ADA level was associated independently with tubercular meningitis as compared to viral meningitis. When we compared tubercular meningitis with bacterial meningitis, we did not find any significantly associated factor (Table S2).

**Table 4:** Analysis of associated risk factors by meningitis type

Characteristics	Bacterial meningitis vs viral meningitis				Tubercular meningitis vs viral meningitis			
	Univariate analysis		Multivariate analysis^#^		Univariate analysis		Multivariate analysis^#^	
	Odds ratio (95% CI)	*P* value	Adjusted OR (95% CI)	*P* value	Odds ratio (95% CI)	*P* value	Adjusted OR (95% CI)	*P* value
Age, 40 years	3.1 (1.18.8)	0.034	2.6 (0.88.9)	0.134	3.9 (0.917.3)	0.078	6.6 (0.2180)	0.265
Male sex	1.8 (0.65.2)	0.291	1.5 (0.45.8)	0.555	0.7 (0.22.9)	0.599	0.2 (0.14.0)	0.297
Place of residence, rural	3.7 (1.310.7)	0.015	4.1 (1.214.4)	0.030	5.7 (1.228.1)	0.032	1.5 (0.120.7)	0.769
Immunocompromised state^a^	2.9 (0.323.8)	0.332			8.0 (0.882.5)	0.081	10.1 (0.4252)	0.159
Duration of fever at admission, 7 days	1.2 (0.43.5)	0.742			2.5 (0.610.9)	0.212		
Neck stiffness	2.8 (1.08.1)	0.062			1.9 (0.48.0)	0.405		
Lab findings								
Leukocytosis (1010^9^/L)	1.5 (0.54.4)	0.423			0.5 (0.12.1)	0.344		
Raised liver enzymes (AST>40 or ALT>35 U/L)	1.8 (0.75.1)	0.244			0.7 (0.22.9)	0.618		
Raised blood urea (>7.1 mmol/L)	2.5 (0.511.9)	0.258			5.3 (0.833.5)	0.076	5.5 (0.2177)	0.336
Raised CRP (1 mg/dL)	3.8 (1.211.7)	0.021			1.8 (0.48.2)	0.447		
CSF analysis								
White blood cells (/L), 100	4.1 (1.411.9)	0.009			4.6 (0.922.3)	0.059		
Neutrophil count (/L), 50	4.1 (1.411.9)	0.009			3.1 (0.713.7)	0.137		
Protein (g/L), 1.0	8.7 (2.629.2)	<0.001			20.6 (3.2133)	0.001		
CSF/blood glucose ratio, 40	27.6 (3.5218.9)	0.002			25.2 (2.5255)	0.006		
ADA (IU/L), 10	26.1 (3.3206)	0.002	25.5 (3.1212)	0.003	40.5 (3.9417)	0.002	42.2 (2.0882)	0.016
Abnormal CT/MRI	0.4 (0.11.8)	0.256			0.2 (0.11.1)	0.070		

^#^In multivariate analysis, variables with *P* value less than 0.2 in univariate analysis were included along with age and sex. Using backward stepwise method, the result of the final model was shown. Variables in CSF analysis were correlated, so we included only variable for ADA in the final multivariate model

^a^This includes diabetes mellitus, liver cirrhosis, HIV positive, renal diseases, cancer, liver diseases, and chronic alcoholic diseases

## Discussion

In this study, we assessed the epidemiological and clinical characteristics of 583 meningitis patients along with their identified etiologies. We found that most common presenting symptoms of CNS infections were headache, fever, and loss of consciousness, and most common signs were neck stiffness and low GCS score. In 27.5% of patients, a definite etiology of CNS infection was identified. The most common pathogens were *S. suis*, *N. meningitidis*, *M. tuberculosis*, *S. pneumoniae*, and HSV. When compared with viral meningitis, bacterial meningitis was independently associated with the rural area of residence and high ADA (10 IU/L); similarly, tubercular meningitis was independently associated with high ADA (10 IU/L).

CSF ADA has been studied as a biomarker for its diagnostic utility for TBM. Various studies show variable sensitivities and specificities of CSF ADA for its diagnostic accuracies, which depend on the cutoff levels and prevalence of the disease in the study population [12,14]. We found that CSF ADA (10 IU/L) was strongly associated with bacterial meningitis and TBM when compared with viral meningitis. The adjusted odds ratio was 42.2 for TBM, which was higher than that of 26.1 for bacterial meningitis. Systematic review and meta-analysis have shown that CSF ADA cannot differentiate TBM from bacterial meningitis; however, it can improve the diagnosis of TBM at ADA level >8 IU/L, particularly when bacterial infection has been ruled out [21]. We had similar observations at 10 IU/L. As ADA assay is inexpensive, easy to perform, and quick to get results, it should be incorporated in the management of CNS infections, especially TBM. TBM presents with non-specific clinical features and available diagnostic tests, including Ziehl-Neelsen staining; GeneXpert MTB/RIF lack sensitivity, and culture of mycobacteria takes time and can only be performed in specialized laboratory which may not be easily available. Therefore, ADA should be used routinely as one of the diagnostic tools while managing CNS infections.

CNS infections have some classical features, including headache, altered mental status, loss of consciousness, and neck stiffness in the background of fever or recent febrile illness [22]. In our study, we found that 84.7% patients with CNS infection had headache, 37.9% had a history of loss of consciousness, 38.4% had neck stiffness, and 38.8% had fever (>38C). Thirty-two percent of patients had GCS scores less than 15. During the study period, 2 patients (0.3%) died, and 89 (15.5%) were discharged to home at comatose state, which shows the detrimental effects of CNS infections. Other previous studies have also shown high mortality and morbidity rates of CNS infection in Vietnam [5, 6]. There could be various reasons for the high morbidity and mortality of CNS infection in Vietnam. Firstly, *S. suis* is the most common cause of CNS infection, which is associated with pigs, and it is common in eating not well-cooked pig products (e.g., raw blood) or rearing pigs at home. Besides, the pig is a host for Japanese encephalitis, which can also cause encephalitis [23, 24]. Regarding preventive measures, although effective vaccines are available at present for other common bacterial infections: *S. pneumoniae* and *N. meningitidis*, these vaccines were not routinely administered to children or adults as part of immunization at the time of this study. Besides, immunocompromised states also increase the risk of CNS infections [25]. In our study population, 16.5% patients were immunocompromised, including 6.9% had diabetes and 6.4% had HIV.

In this hospital, if patients have a history or examination findings of opportunistic infections, which indicate immunocompromised states or HIV infection, or patients need an invasive treatment, we screen them for HIV testing. In the current analysis, we presumed most of HIV not-tested groups were not HIV infected. Among 583 patients with CNS infections, 60.9% were tested for HIV. When we compared the HIV tested with HIV not-tested groups, we found that male sex, age <45 years, hepatomegaly, and hospital admission duration over 7 days were independently associated with HIV-tested patients. Among HIV-positive cases, the prevalence of HIV is higher in male sex and young people than female sex and older people in Vietnam [26]. We found that 14.1% of HIV-tested patients had hepatomegaly. This is higher than the prevalence of hepatomegaly (4%) found among HIV patients presenting in outpatient clinics in Ho Chi Minh City, Vietnam [27]. The higher proportion of hepatomegaly might be due to the effects of other systemic infections, such as tuberculosis. The longer hospital stay in this population could be explained by the increased severity of illness [28, 29], and they tended to be referred to another hospital more frequently than the HIV not-tested patient group.

Burden of the disease, etiologies, and morbidity and mortality of CNS infection vary according to various factors, including age of the patients and geographical regions [30]. The disease incidence is high in sub-Saharan Africa and South East Asia. Regarding the common etiologies of bacterial meningitis, *N. meningitis*, *S. pneumoniae*, *H. influenzae*, and *Salmonella spp*. are common bacterial pathogens detected in Africa [31, 32], and *S. pneumoniae*, *N. meningitidis*, *Group B streptococcus*, *K. pneumoniae*, *S. viridans*, and *S. suis* are common in Asia [33,35]. Case fatality rate of bacterial meningitis was found to be highest (32.7%) in Swaziland and lowest (2.4%) in Singapore [30]. In our study, we found that *S. suis* was the commonest cause of bacterial meningitis, followed by *N. meningitidis* and *S. pneumoniae*. Our findings are consistent with the findings of previous research in Vietnam [5, 6]. *S. suis* is a zoonotic disease, and it is associated with exposure (contact) to pigs or pork. Eating undercooked pig products is a common practice in Vietnam and is found to be associated with *S. suis* infection [16]. Among viral meningitis, we found HSV was the most common followed by echoviruses. The viral etiologies were similar to the previous report from Hanoi [5], although we could not perform the serological diagnosis of some viral pathogens for CNS infections, such as Japanese encephalitis and dengue virus. JE and dengue virus were some of the common etiologies detected in previous studies in Vietnam [6, 36]. TBM was one of the common pathogens detected in this study. We detected 13 individual TBM cases and 2 cases with *H. influenzae* in 408 patients with CNS infections. HIV positivity was associated with TBM. As we referred all TB meningitis cases, once they were diagnosed, to another special hospital for TB treatment, we did not know the outcome of these patients. TBM is a severe form of TB with high morbidity and mortality. Patients with HIV are at 20 times higher risk of developing TB [37]. A study in Vietnam showed that the mortality rate of TBM cases was more than twice in HIV-positive patients compared to HIV-negative patients (65% vs 30%) [38]. Diagnosis of TBM is difficult, and to differentiate it from bacterial or viral meningitis by clinical features only is often impossible [7]. Microscopy of CSF has low sensitivity, and growth in conventional culture is slow. Early diagnosis of TBM is vital to decrease the morbidity and mortality as the early diagnosis helps to start the antitubercular drugs on time. However, there is a lack of highly sensitive and specific diagnostic assay for TBM [37]. Although GeneXpert MTB/Rif has improved the diagnosis of pulmonary tuberculosis, its sensitivity to diagnose TBM has been variable because of the limitation of the PCR-based tests and the low bacterial density in CSF samples [39]. Therefore, there is an urgent need to develop a highly sensitive and specific diagnostic test for TBM.

Comparing the characteristics among the viral, bacterial, and tubercular meningitis, we found that the median age of patients with viral meningitis was significantly lower than patients with bacterial meningitis or TBM. The age distribution seemed similar to the previous study carried out in Vietnam, except for the TBM patients who tended to be older in our study population [5]. The proportion of patients from the rural area was higher than urban area in bacterial meningitis and TBM, which was quite opposite in viral meningitis. Rural place of residence was independently associated with bacterial meningitis when compared with viral meningitis. One of the reasons of this association is likely due a higher proportion of exposure to pig in rural area (OR 2.1 (95% CI 0.59.9)), although the number of people exposed to pig was small. However, our data showed a strong association between patients with *S. suis* and history of exposure to pig (*P*<0.001); other probable reasons are people in rural areas tend to be poorer, with low hygiene, and malnourished so that they have higher susceptibility of bacterial infections, including tuberculosis [40, 41]. Laboratory investigations showed that the median white blood cells were significantly less in TBM than viral or bacterial meningitis. The proportion of patients with raised CRP level (1 mg/dL) was higher in bacterial meningitis than viral and TBM. These findings were similar to those of the previous studies [5, 42].

This study has limitations. We could not do the serological tests for viral etiologies, which had limited the detections of some of the viruses, including Japanese encephalitis. Some of the CSF samples with abnormal cells or protein were not available for PCR. The amount of CSF in some samples was not adequate to do culture properly that may diminish the identification of some bacterial pathogens. Culture of *Mycobacterial tuberculosis* was also not available in the hospital laboratory. As this is the tertiary level hospital, most of the patients were very serious and were referred from the other hospital that led to a higher proportion of not-recovered or comatose discharge. We could not determine the outcome of TBM patients as we had to refer them to TB special hospital once diagnosed. Due to low sample size, we could not detect any significant differences in characteristics of tubercular meningitis and bacterial meningitis.

## Conclusions

Using the molecular diagnostic method in addition to the conventional culture of CSF, we detected a range of viral, bacterial, mycobacterial, and fungal pathogens of CNS infections in adult patients. Addition of molecular methods had improved the identification of pathogens. Rural residence was associated with bacterial meningitis. CSF ADA 10 IU/L was strongly associated with bacterial meningitis and TBM. Once bacterial meningitis is ruled out, it may be helpful to diagnose TBM.

## Supplementary Information

**Additional file 1: Table S1.** Comparison of epidemiological and clinical characteristics between HIV-not-tested and HIV-tested population.

**Additional file 2: Table S2.** Analysis of associated risk factors of tubercular meningitis versus bacterial meningitis.

**Additional file 3.**

## Data Availability

De-identified data of this study will be available at the reasonable request to the corresponding author.

## Abbreviations

ADA: Adenosine deaminase
CMV: Cytomegalovirus
CNS: Central nervous system
CRP: C-reactive protein
CSF: Cerebrospinal fluid
GCS: Glasgow Coma Scale
HIV: Human immunodeficiency virus
HSV: Herpes simplex virus
TB: Tuberculosis
TBM: Tubercular meningitis
WBC: White blood cell
