# Supplementary material for: Characteristics and biomarkers of patients with central nervous system infection admitted to a referral hospital in Northern Vietnam
Source: Trop Med Health. 2021 May 21;49:42. doi: 10.1186/s41182-021-00322-2 (PMC8139123; doi:10.1186/s41182-021-00322-2)
Supplement: Supplementary file 1 — Additional file 1: Table S1. Comparison of epidemiological and clinical characteristics between HIV-not-tested and HIV-tested population. [file 41182_2021_322_MOESM1_ESM.docx]

Table S1. Comparison of epidemiological and clinical characteristics between HIV-not-tested and HIV-tested population

| Characteristics | HIV-not-tested  N=228 (%) | HIV-tested  N=355 (%) | Odds ratio  (95% CI) | *P* value | Adjusted Odds ratio (95% CI) | *P* value |
| --- | --- | --- | --- | --- | --- | --- |
| Age, <45 years | 104 (45.6) | 187 (52.7) | 1.33 (0.95-1.85) | 0.096 | 1.85 (1.27-2.69) | 0.001 |
| Male sex | 126 (55.3) | 239 (67.3) | 1.67 (1.18-2.35) | 0.003 | 1.70 (1.17-2.47) | 0.005 |
| Geography of residence  Flatland  Mountain  Coastal area | 181 (79.4)  22 (9.6)  25 (11.0) | 263 (74.1)  47 (13.2)  45 (12.7) | Ref  1.47 (0.86-2.52)  1.24 (0.73-2.09) | Ref  0.162  0.423 | 1.64 (0.92-2.93)  1.23 (0.69-2.20) | 0.095  0.474 |
| Place of residence  Urban  Rural | 65 (28.5)  163 (71.5) | 115 (32.4)  240 (67.6) | 1.20 (0.84-1.73)  Ref | 0.322 | 0.80 (0.53-1.19)  Ref | 0.269 |
| Exposure to animal, pig | 6 (2.6) | 9 (2.5) | 0.96 (0.34-2.74) | 0.943 |  |  |
| Immunocompromised state  Diabetes  Cirrhosis  Cancer | 17 (7.5)  2 (0.9)  3 (1.3) | 23 (6.5)  11 (3.1)  3 (0.9) | 0.86 (0.45-1.65)  3.61 (0.79-16.5)  0.64 (0.13-3.19) | 0.649  0.097  0.586 |  |  |
| Antibiotic use before hospitalization | 50 (21.9) | 105 (29.6) | 1.5 (1.01-2.20) | 0.042 | 1.51 (0.99-2.32) | 0.058 |
| Referred from other hospitals | 144 (63.2) | 242 (68.2) | 1.25 (0.88-1.77) | 0.212 |  |  |
| Fever (>38°C) | 83 (37.4) | 141 (39.7) | 1.10 (0.78-1.56) | 0.576 |  |  |
| Duration of fever at admission, ≥7 days | 42 (18.4) | 129 (36.3) | 2.53 (1.70-3.77) | <0.001 | 2.25 (1.45-3.50) | <0.001 |
| Headache | 197 (86.4) | 297 (83.7) | 0.81 (0.50-1.29) | 0.370 |  |  |
| History of convulsion | 11 (4.8) | 27 (7.6) | 1.62 (0.79-3.34) | 0.188 |  |  |
| History of loss of consciousness | 67 (29.4) | 154 (43.4) | 1.84 (1.29-2.62) | 0.001 | 1.71 (0.86-3.37) | 0.124 |
| Neck stiffness | 82 (36.0) | 142 (40.0) | 1.19 (0.84-1.67) | 0.329 |  |  |
| Rash | 25 (11.0) | 45 (12.7) | 1.18 (0.70-1.98) | 0.535 |  |  |
| Hypoxemia | 9 (4.0) | 25 (7.0) | 1.84 (0.84-4.02) | 0.125 |  |  |
| Hypotension | 2 (0.9) | 2 (0.6) | 0.64 (0.09-4.58) | 0.657 |  |  |
| Hepatomegaly | 13 (5.7) | 50 (14.1) | 2.71 (1.44-5.11) | 0.002 | 2.45 (1.25-4.81) | 0.009 |
| Splenomegaly | 3 (1.3) | 7 (2.0) | 1.50 (0.39-5.89) | 0.554 |  |  |
| Glasgow Coma Scale  15  8 to 14  <8 | 171 (76.0)  52 (23.1)  2 (0.9) | 221 (62.8)  119 (33.8)  12 (3.4) | Ref  1.77 (1.20-2.59)  4.64 (1.03-21.0) | 0.003  0.046 | Ref  0.97 (0.47-1.99)  3.33 (0.64-17.3) | 0.926  0.154 |
| Hospital duration, ≥7 days | 162 (71.1) | 263 (74.3) | 1.18 (0.81-1.70) | 0.390 |  |  |
| Outcomes |  |  |  |  |  |  |
| Complete recovery | 67 (29.4) | 64 (18.0) | Ref |  | Ref |  |
| Referral to another hospital | 47 (20.6) | 107 (30.1) | 2.38 (1.47-3.87) | <0.001 | 1.73 (1.00-2.99) | 0.050 |
| Incomplete recovery | 86 (37.7) | 121 (34.1) | 1.47 (0.95-2.29) | 0.085 | 1.41 (0.88-2.26) | 0.150 |
| Not recovered-discharged to home | 28 (12.3) | 61 (17.2) | 2.28 (1.30-4.0) | 0.004 | 1.37 (0.74-2.57) | 0.319 |
| Death | 0 (0.0) | 2 (0.6) | - | - | - | - |
