# Supplementary material for: Characteristics and biomarkers of patients with central nervous system infection admitted to a referral hospital in Northern Vietnam
Source: Trop Med Health. 2021 May 21;49:42. doi: 10.1186/s41182-021-00322-2 (PMC8139123; doi:10.1186/s41182-021-00322-2)
Supplement: Supplementary file 2 — Additional file 2: Table S2. Analysis of associated risk factors of tubercular meningitis versus bacterial meningitis. [file 41182_2021_322_MOESM2_ESM.docx]

Table S2. Analysis of associated risk factors of tubercular meningitis versus bacterial meningitis

| Characteristics | Odds ratio (95% CI) | *P* value |
| --- | --- | --- |
| Age, ≥40 years | 1.2 (0.3-4.4) | 0.736 |
| Male sex | 0.4 (0.1-1.3) | 0.120 |
| Place of residence, rural | 1.5 (0.4-6.1) | 0.544 |
| Immunocompromised state* | 2.8 (0.7-10.8) | 0.136 |
| Duration of fever at admission,  ≥7 days | 2.1 (0.6-6.9) | 0.219 |
| Neck stiffness | 0.7 (0.2-2.2) | 0.506 |
| Lab findings  Leukocytosis (≥10x10^9^/L) | 0.3 (0.1-1.0) | 0.067 |
| Raised liver enzymes  (AST>40 or ALT>35 U/L) | 0.4 (0.1-1.3) | 0.118 |
| Raised blood urea (>7.1 mmol/L) | 2.1 (0.6-7.5) | 0.230 |
| Raised CRP (≥1 mg/dL) | 0.5 (0.1-1.7) | 0.253 |
| CSF analysis  White blood cells (/µL), ≥100 | 1.1 (0.3-4.5) | 0.882 |
| Neutrophil count (/µL), ≥50 | 0.8 (0.2-2.7) | 0.663 |
| Protein (g/L), ≥1.0 | 2.4 (0.5-11.6) | 0.286 |
| CSF/blood glucose ratio, ≤40 | 0.9 (0.3-3.2) | 0.884 |
| ADA (IU/L), ≥10 | 1.6 (0.4-5.5) | 0.496 |
| Abnormal CT/MRI | 0.4 (0.1-1.8) | 0.246 |

*This includes diabetes mellitus, liver cirrhosis, HIV positive, renal diseases, cancer, liver diseases, and chronic alcoholic diseases.
