# Supplementary material for: Characteristics and biomarkers of patients with central nervous system infection admitted to a referral hospital in Northern Vietnam
Source: Trop Med Health. 2021 May 21;49:42. doi: 10.1186/s41182-021-00322-2 (PMC8139123; doi:10.1186/s41182-021-00322-2)
Supplement: Supplementary file 3 — Additional file 3. [file 41182_2021_322_MOESM3_ESM.pdf]

ANNEX 1

Clinical Information Sheet

Patient ID

1. Sample information

	Sample number	Date of Collection (dd/mm/yyyy)
1st blood sample	A......................	/ /
2nd blood sample	B.....................	/ /
CSF	C......................	/ /
Others	D......................	/ /

2. Patient basic information

Age			
Sex	M □ / F □		
Province of residence		District	
Occupation			
Date of admission (dd/mm/yyyy)	/ /		
Date of discharge (dd/mm/yyyy)	/ /		

3. Pre-hospital information

Regular medication	N□	Y	
Underlying disease	N□	Y	diabetes□, heart disease□, liver disease□, renal disease□(hemodyalisis□), cerebrovascular disease□, cancer□, others........................
Vacctination	N□	Y	diphteria□, tetanus□, pertusis□, measles□, rubella□, BCG□, HBV□, Meningococcal□, Hib□, typhoid□, others..........................
Alcohol intake	N□	Y	everyday□, sometime□
Smoking	N□	Y	............../ pack-year
Significant previous illness	N□	Y	....
Referral from other hospitals	N□	Y	which hospital..

4. Epidemiological information

Residence	central Hanoi□, not central Hanoi□, outside Hanoi□			
	urban□, rural□			
	flatland□, mountainous□, coastal□			
Animal contact (within 1month)<br>Pet, livestock, many seen around house	N□	Y	pig□, chicken□, rat □, dog□, cat□,<br>others............................	
Insect bite (within 1 month)	N□	do not know□	Y	tick□, mite□, others...............
Travel history (within 1 month)	N□	Y	where...	
Similar symptom in family	N□	Y	who..	

5. Clinical manifestation

Duration of fever before admission (days)	
Onset of fever<br>Sudden; over 1-2 days<br>Progressive; over 3-5 days	sudden□, gradual□
Character of fever<br>continous; high temp. without falling<br>intermittent; sometimes normal temp.<br>periodical; repeating every several days	continuous□, intermittent□, periodical□

Any symptoms below during the period from onset to admission?

headache	N□	Y□	abdominal pain	N□	Y□
sore throat	N□	Y□	nausea/vomiting	N□	Y□
myalgia	N□	Y□	back pain	N□	Y□
rigor	N□	Y□	joint pain	N□	Y□
chest pain	N□	Y□	loss of consciousness	N□	Y□
cough	N□	Y□	mental change	N□	Y□

6. Physical findings around admission

Blood pressure	/ mmHg
Heart rate (pulse rate)	/min
Respiratory rate	/min
SpO2	%
Consciousness	Glasgow coma scale.....................

Structure	small□, moderate□, tall□			fat□, thin□
Injected conjunctiva	N□	Y□		
Anemic conjunctiva	N□	Y□		
Icteric conjunctiva	N□	Y□		
Injected throat	N□	Y□		
Lung rale	N□	Y	right□, left□, both□	wheeze□, crackle□
Heart murmur	N□	Y	systolic□, diastolic□	
Abdominal tenderness	N□	Y	right upper□, left upper□, right lower□, left lower□	
Abdominal distention	N□	Y□		
Hepatomegaly	N□	Y□		
Splenomegaly	N□	Y□		
CVAknocking tenderness	N□	Y	right□, left□, both□	
Edema	N□	Y	leg□, face□, others............................	
Skin congestion	N□	Y□		
Rash	N□	Y	body□, legs□,<br>others...............	macular□, papular□,<br>maculopapular□, blister□,<br>hemorrhagic□, erythema□
Eschar	N□	Y	body□, legs□,<br>others.............	Number; 1□, 2 or more□
Lymphnode swelling	N□	Y	neck□, axilla□,<br>inguinale□	Number; 1□, 2 or more□
Joint swelling	N□	Y	knee□, ankle□, elbow□, wrist□, others...........	
Calf tenderness	N□	Y□		
Neck stiffness	N□	Y□		
Paralysis	N□	Y	right□, left□, others.................	

7. Laboratory data and imaging

1st basic routine lab data after admission			date (................/................../.....................)		
WBC		ALP		Na	
Neu		Tbil		K	
Hb		Dbil		Cl	
Ht		TP		Ca	
Plt		Alb		Glucose	
sGOT		UN		CRP	
sGPT		Creatinine			

Further lab data					
HIV	positive□, negative□, not checked□			CD4 count	
Cultures	blood□, sputum□, urine□, CSF□, others□.........			result	
CSF analysis	date....../......./.........	protein..................., glucose................., cells............... (neutro............%, lymph............%)			
Serology	for..................	blood□, CSF□	date......../........./.........	result	
PCR	for..................	blood□, CSF□, sputum□, others□.............		result	
ANA		Other tests			
Biopsy	date......../........../............	specimen		result	

Imaging study				
Chest X ray	normal□	infiltrate□, consolidation□	single□, multiple□	R□, L□
Abdominal US	normal□	result		
CT (......./......../......)	chest□, brain□, abdominal□, others□	normal□, result..		
MRI (......./......./.......)	brain□, others□	normal□, result..		

8. Final diagnosis

N□	Y; clinical diagnosis□.............................., confirmed diagnosis□..................................

9. Treatment

1st Antibiotics	from....../......./..........			g/day		days
2nd Antibiotics	from....../......./..........			g/day		days
3rd Antibiotics	from....../......./..........			g/day		days
Steroid	from....../......./..........			mg/day		days
Other Tx	from....../......./..........					

10. Hospital course

Patient status	recovery□, sequelae□, died□, discharge w/o recovery□
Defervescence	date ..../................./....................
Disappearance of rash	date ..../................./.....................
Drug adverse effects	N□, Y..
